# Significance of Plankton Community Structure and Nutrient Availability for the Control of Dinoflagellate Blooms by Parasites: A Modeling Approach

**DOI:** 10.1371/journal.pone.0127623

**Published:** 2015-06-01

**Authors:** Catharina Alves-de-Souza, David Pecqueur, Emilie Le Floc’h, Sébastien Mas, Cécile Roques, Behzad Mostajir, Franscesca Vidussi, Lourdes Velo-Suárez, Marc Sourisseau, Eric Fouilland, Laure Guillou

**Affiliations:** 1 Laboratório de Ficologia, Departamento de Botânica, Museu Nacional/Universidade Federal do Rio de Janeiro, Quinta da Boa Vista S/N, São Cristóvão, Rio de Janeiro, RJ, Brasil; 2 UMR 9190 MARBEC Center of Marine Biodiversity, Exploitation & Conservation, CNRS, UM, IRD, IFREMER, Université Montpellier, Place E. Bataillon, CC 093, Montpellier, France; 3 UMS 3282 OSU OREME-MEDIMEER, IRD, UM, CNRS, SMEL, 2 rue des Chantiers, Sète, France; 4 UMR 9190 MARBEC Center of Marine Biodiversity, Exploitation & Conservation, CNRS, UM, IRD, IFREMER, SMEL, Sète, France; 5 Ifremer, DYNECO PELAGOS, Plouzané, France; 6 CNRS, UMR 7144 & Université Pierre et Marie Curie, Station Biologique de Roscoff, Place Georges Teissier, Roscoff, France; University of Connecticut, UNITED STATES

## Abstract

Dinoflagellate blooms are frequently observed under temporary eutrophication of coastal waters after heavy rains. Growth of these opportunistic microalgae is believed to be promoted by sudden input of nutrients and the absence or inefficiency of their natural enemies, such as grazers and parasites. Here, numerical simulations indicate that increasing nutrient availability not only promotes the formation of dinoflagellate blooms but can also stimulate their control by protozoan parasites. Moreover, high abundance of phytoplankton other than dinoflagellate hosts might have a significant dilution effect on the control of dinoflagellate blooms by parasites, either by resource competition with dinoflagellates (thus limiting the number of hosts available for infection) or by affecting numerical-functional responses of grazers that consume free-living parasite stages. These outcomes indicate that although both dinoflagellates and their protozoan parasites are directly affected by nutrient availability, the efficacy of the parasitic control of dinoflagellate blooms under temporary eutrophication depends strongly on the structure of the plankton community as a whole.

## Introduction

Most harmful algal blooms (HABs) are formed by toxic or otherwise harmful dinoflagellate species that have potentially devastating effects on marine habitats including the closure of shellfish beds, massive fish kills, and death of other marine fauna [1]. They are frequently initiated in semi-confined areas like harbors, lagoons, and estuaries where sudden nutrient pulses from rivers draining urban centers and agricultural watersheds result in temporary eutrophication [2–5]. Such increases in nutrient availability could promote bloom formation not only by boosting dinoflagellate growth rates (bottom-up control) [6], but also by changing the structure and dynamics of plankton communities, including grazers and parasites that are natural enemies of dinoflagellates (top-down control) [7–9]. Amongst the top-down mechanisms influencing dinoflagellate blooms, parasitism stands out as one of the most direct and widespread [10]. Yet, little is known about how shifts in plankton community composition driven by changes in nutrient concentration could affect host-parasite dynamics in plankton systems.

*Amoebophrya* (Alveolata, Syndiniales) is a genus of obligate parasitic eukaryotes infecting a wide range of marine dinoflagellates [10]. The life-cycle of these parasites is characterized by an alternation between a biflagellated free-living infective stage (the dinospore) and an intracellular stage (the trophont). Once inside the host, maturation takes 2–3 days and eventually culminates with the death of the host and the release of a motile worm-shaped multinucleated and multiflagellated structure (the vermiforme), which fragments within a few hours, releasing hundreds of dinospores, each potentially capable of infecting a novel host [11, 12]. This simple life-cycle, combined with a short generation time, make *Amoebophrya* spp. excellent models for studying host-parasite dynamics within plankton communities.

Both field observations and model predictions indicate that these highly virulent parasites have the capacity to efficiently control blooms of their dinoflagellate hosts [10, 13–15]. However, parasite and host populations do not exist as isolated pairs, but rather as parts of multispecies systems. In fact, dinoflagellates could be simultaneously affected by other biotic interactions, such as competition for nutrient uptake with other phytoplankton and grazing by microzooplankton [6, 16]. In addition, grazing on free-living parasite stages can significantly decrease parasite infections in plankton systems [9, 17]. Thus, in order to understand the factors affecting parasitic control of dinoflagellate blooms, it is important to consider the structure of the whole plankton community, including both the specific composition and the relative abundance of their members.

Here, the relative importance of nutrient availability and biotic interactions (i.e., parasitism, grazing and competition for nutrients) to dinoflagellate bloom dynamics was evaluated by numerical simulations. The main goal was to assess how changes in the relative abundance of host and non-host species driven by changes in nutrient concentration could affect the dilution effect on parasite infections. Model outcomes indicate that although increasing nutrient concentration promotes both the growth of dinoflagellates and the development of their protozoan parasites, the efficacy of parasitic control of dinoflagellate blooms under temporary eutrophication depends strongly on the structure of the plankton community as a whole.

## Materials and Methods

### Modeling approach

The model was based upon previous mathematical approaches related to *Amoebophrya-*host dynamics [15, 18]. In order to avoid the inclusion of too many trophic links (which would lead to an unnecessary complexity), the modeled community included as few components as possible. Thus, we considered a simple plankton community constituted by eukaryotes, only, including dinoflagellates, their parasites, their potential competitors (phytoplankton other than dinoflagellates), as well as their grazers (microzooplankton). Although mesozooplankton can exhibit high grazing rates on blooming dinoflagellates [19], they were not considered because their long generation times reduce the likelihood of exerting immediate control over phytoplankton blooms following nutrient pulses [20]. Nitrate concentration was chosen as the forcing variable because nitrogen is generally the most limiting nutrient in marine systems [5]. Thus, it would be reasonable to consider it as the main nutrient to take into account when considering a situation of temporary eutrophication, in which a phytoplankton community is suddenly exposed to an increased concentration of nutrients. Moreover, this is the nutrient with the largest volume of available information in the literature on nutrient uptake, which facilitated the parameterization of the model regarding phytoplankton growth.

Briefly, the phytoplankton community was represented in the model by nanophytoplankton other than diatoms and dinoflagellates (*A*), diatoms (*D*), uninfected (*H*) and infected (*I*) dinoflagellates. For modeling purposes, phytoplankton were considered to be divided in nanophytoplankton, diatoms and dinoflagellates with no overlapping between those groups, although the term nanophytoplankton usually refers to phytoplankton smaller than 20 μm that may include small diatoms and dinoflagellates. Rotifers (*R*) grazed only on prey smaller than 20 μm [21], i.e. nanophytoplankton (*A*) and dinospores (*P*) (the free-living parasite stages). Although microciliates (*C*) can have a wide prey range [22], modeled microciliates grazed only on diatoms and dinoflagellates. By assigning different preys to microciliates and rotifers, we were able to evaluate the individual importance of these two grazers for the host-parasite dynamics.

Parameters/state variables and equations used in the simulations are shown in Tables 1 and 2, respectively. The initial values of the variables reported in Table 1 were based on the specific composition of the eukaryotic plankton community observed at the beginning of a bloom of the dinoflagellate *Prorocentrum triestinum* (infected by *Amoebophrya* spp.) following an experimental nutrient pulse obtained during a mesocosm experiment performed in Thau Lagoon (southern France) in autumn 2009. Similarly, the NO_3_+NO_2_ values observed before (1 μM) and after (36 μM) the experimental nutrient pulse were used here as the nitrate concentrations for running the simulations under oligotrophic and eutrophic conditions, respectively. The choice of the plankton community from Thau Lagoon as a template for the model is justified because this system is periodically subject to dinoflagellate blooms [23] and temporary eutrophication following river discharge [24]. More details regarding the methods used in the determination of these variables as well as a short description of the composition of the plankton community in Thau Lagoon, are available in the S1 Appendix (Sections S1-1 and S1-2). No specific permissions were required for these locations/activities and these field collections did not involve endangered or protected species.

**Table 1:** Values of parameters and state variables considered in the numerical simulations.

Parameter/State variable		Unit	Values of parameters/Initial values of state variables	Source
Symbol	Name			
	**Dinoflagellates**			
*H*	uninfected abundance	cells L^–1^	1.48 × 10^5^	(this study)^a^
*I*	infected abundance	cells L^–1^	3 × 10^3^	(this study)^a^
*r_h_*	maximal growth rate	d^–1^	0.7	[25]^b^
*K_h_*	half saturation constant for N uptake	μM	2.3	[26]^c^
*Q_h_*	N cellular quota	μM	7.12 × 10^–7^	[27]^d^
	**Parasites**			
*P*	abundance of dinospores (free-living parasite stages)	cells L^–1^	1.16 × 10^5^	(this study)
*ε*	number of dinospores released per infected host	dinospores host^–1^	150	(this study)
*m*	mortality rate	d^–1^	0.26	[12]^e^
*a*	search rate	L dinospore^–1^ d^–1^	1.34 × 10^–8^	[18]^e^
*h*	handling time	d^–1^	2.46	[12]^e^
	**Diatoms**			
*D*	diatom abundance	cells L^–1^	2.5 × 10^4^	(this study)
*r_d_*	maximal growth rate	d^–1^	1.5	[28]^f^
*K_d_*	half saturation constant for N uptake	μM	1.2	[26]^g^
*Q_d_*	N cellular quota	μM	6.12 × 10^–7^	[29]^h^
	**Nanophytoplankton**			
*A*	nanophytoplankton abundance	cells L^–1^	1.9 × 10^6^	(this study)
*r_a_*	maximal growth rate	d^–1^	0.7	[30]^i^
*K_a_*	half saturation constant for N uptake	μM	0.5	[31]^j^
*Q_a_*	N cellular quota	μM	4.33 × 10^–9^	[29]^k^
	**Microciliates**			
*C*	microciliate abundance	cells L^–1^	3.2 × 10^3^	(this study)
*r_c_*	growth rate	d^–1^	Eq. (11) (Table 2)	[32]^l^
*r_cmax_*	maximal growth rate	d^–1^	0.32	[32]^l^
*K_rc_*	constant sustaining ½ r*_cmax_*	preys L^–1^	1.8 × 10^6^	[32]^l^
*x’_c_*	threshold for ciliate growth	preys L^–1^	7.24 × 10^5^	[32]^l^
*G_c_*	grazing rate	preys ciliate^–1^ d^–1^	Eq. (12) (Table 2)	[32]^l^
*G_cmax_*	maximal ingestion rate	preys ciliate^–1^ d^–1^	168	[32]^l^
*K_Gc_*	constant sustaining ½ G*_cmax_*	preys L^–1^	3.26 × 10^7^	[32]^l^
	**Rotifers**			
*R*	rotifer abundance	ind L^–1^	20	(this study)
*r_r_*	growth rate	d^–1^	Eq. (14) (Table 2)	[33]^m^
*r_rmax_*	maximal growth rate	d^–1^	1.03	[33]^m^
*K_rr_*	constant sustaining ½ r*_rmax_*	preys L^–1.^	4.74 × 10^6^	[33]^m^
*x’_r_*	threshold for rotifer growth	preys L^–1^	2.52 × 10^6^	[33]^m^
*G_r_*	grazing rate	preys rotifer^–1^ h^–1^	Eq. (15) (Table 2)	[34]
*G_rmax_*	maximal ingestion rate	preys rotifer^–1^ h^–1^	2.7 × 10^3^	[34]
*K_Gr_*	constant sustaining ½ G*_rmax_*	preys L^–1^	1.59 × 10^8^	[34]

^a^Only *Prorocentrum triestinum* was considered because this species contributed to 99% of the total dinoflagellate abundance in Thau Lagoon (S1 Appendix, section S1-2).

^b^Maximal growth rate of *P*. *triestinum*.

^c^Average value of half saturation constants for nitrate uptake of dinoflagellate species presented in Table 3.7 of this author.

^d^Nitrogen cell quota of *Prorocentrum micans*.

^e^Parameters of *Amoebophrya* sp. infecting *Karlodinium micrum*.

^f^Average value of the mean doubling rates (d^–1^) of *Leptocylindrus minimus*, *Leptocylindrus danicus*, *Cylindrotheca closterium* and *Thalassionema nitzschioides* (the dominant diatoms species in Thau Lagoon; S1 Appendix, section S1-2).

^g^Half saturation constant for nitrate uptake of *Pseudo-nitzschia* sp. shown in Table 3.7 of this author.

^h^Average values of nitrogen cell quota of diatom species presented in Table 1 of these authors.

^i^Average value of nanophytoplankton growth rates (d^–1^) presented by these authors in their Fig 4 (only control experiments, with no nutrient addition, were considered).

^j^Value for nanophytoplankton natural assemblages in Thau lagoon presented by these authors.

^k^Average values of nitrogen cell quota of nanoplankton species presented in Table 1 of these authors.

^l^Average values of *Tiarina fusum* feeding on *Lingulodinium polyedrum* and *Scrippsiella trochoidea* (values in ng C^–1^ were transformed to cells L^–1^ considering carbon content per cell) given by the authors.

^m^Based on average values estimated from growth rates of *Brachionus plicatilis*, *Brachionus rotundiformis* and *Brachionus* sp. feeding on *Tetraselmis suecica* and *Nannochloris atomus* (prey concentrations presented in ng C^–1^ were converted to cells L^–1^ by considering the cellular carbon content of a cell with equivalent spherical diameter of 10 μm and the equation for carbon conversion given by [29]).

**Table 2:** Differential equations used in the numerical simulations.

Equation number	Equation	Source
1	$\frac{\text{d}H}{\text{d}t}=r_{h}Hf_{h}-\frac{aH}{1+ahH}P-\frac{CG_{c}H}{H+I+D}$	[18]^a^ ^,^ ^b^
2	$f_{h}=\frac{N}{K_{h}+N}$	
3	$\frac{\text{d}I}{\text{d}t}=\frac{aH}{1+ahH}P-\frac{I}{h}-\frac{CG_{c}I}{H+I+D}$	[18]^b^
4	$\frac{\text{d}P}{\text{d}t}=\varepsilon \frac{I}{h}-\frac{aH}{1+ahH}P-mP-\frac{RG_{r}P}{P+A}$	[18]^b^
5	$\frac{\text{d}D}{\text{d}t}=r_{d}Df_{d}-\frac{CG_{c}D}{H+I+D}$	[15]^a^
6	$f_{d}=\frac{N}{K_{d}+N}$	
7	$\frac{\text{d}A}{\text{d}t}=r_{a}Af_{a}-\frac{RG_{r}A}{A+P}$	[15]^a^ ^,^ ^b^
8	$f_{a}=\frac{N}{K_{a}+N}$	
9	$\frac{\text{d}C}{\text{d}t}=r_{c}C$	[32]^c^
10	$\frac{\text{d}N}{\text{d}t}=-Hr_{h}f_{h}Q_{h}-Hr_{d}f_{d}Q_{d}-Hr_{a}f_{a}Q_{a}$	
11	$r_{c}=\frac{r_{c\max}\times \left[\left(H+I+D\right)-x{'}_{c}\right]}{K_{rc}+\left[\left(H+I+D\right)-x{'}_{c}\right]}$	[32]^c^
12	$G_{c}=\frac{G_{c\max}\times \left(H+I+D\right)}{K_{Gc}+\left(H+I+D\right)}$	[32]
13	$\frac{\text{d}R}{\text{d}t}=r_{r}C$	[33]^d^
14	$r=\frac{r_{r\max}\times \left[\left(A+P\right)-x{'}_{r}\right]}{K_{rr}+\left[\left(A+P\right)-x{'}_{r}\right]}$	[33]^d^
15	$G=\frac{G_{r\max}\times \left(A+P\right)}{K_{Gr}+\left(A+P\right)}\times \frac{0.94}{1+219000\times T^{-4.35}}\times 24$	[34]^e^

Meanings of the symbols are the same as indicated in Table 1.

^a^Equations modified to include growth based on nutrient uptake following a Michaelis-Menten-Monod function.

^b^Equations modified to include grazing by microciliates or rotifers.

^c^Maximum growth rate was determined at 19°C and was temperature-corrected to 20°C assuming a *Q*_10_ of 2.

^d^Hourly rates were converted to daily rates, assuming a constant growth over 24h.

^e^ *T* is the temperature (20°C).

### Sensitivity analysis

The Sobol' method [35] was used to conduct the sensitivity analysis of the proposed model. This analysis is a global approach to estimate the effect of one parameter on the model output when all other parameters vary, enabling the identification of interactions in the model [36]. Sobol’s first and total order sensitivity indices were estimated for 30-d simulations using the SBtoolbox [37] using Matlab (MathWorks).

### Numerical simulations

Different scenarios representing an increasing degree of community complexity were simulated: i) parasites and dinoflagellates only, ii) parasites, dinoflagellates and grazers, and iii) parasites, dinoflagellates, grazers and other phytoplankton (i.e., nanophytoplankton and diatoms). In each scenario, simulations were run for 30 days under two trophic conditions: oligotrophic (1 μM nitrate) and eutrophic (36 μM nitrate). A 30-d period was chosen because this represents a period in which both the development and the demise of dinoflagellate blooms are expected to occur in Thau Lagoon [38].

Dinoflagellate growth was further modeled, in the presence and absence of parasites, under a series of abundances of phytoplankton other than dinoflagellates (10^4^, 10^5^, 10^6^, 10^7^, 10^8^, 10^9^ cells L^–1^) and increasing concentrations of nitrate (0.5, 1, 5, 10, 20, 30, 40 and 50 μM). In all simulations, the proportion between nanophytoplankton and diatoms was maintained at 99:1. The abundances of dinoflagellates (250,000 cells L^–1^), microciliates (3,200 cells L^–1^) and rotifers (20 ind L^–1^) were fixed. For simplicity, simulations with the presence of parasites started with no infected dinoflagellates and with a dinospore:host proportion of 2:1 (i.e., dinospores = 125,000 cells L^–1^).

The effect of each component of the modeled plankton community on the parasite-dinoflagellate dynamics was assessed by performing additional 30-d simulations where the different components were excluded from the model (one at a time). Negative effects were divided into two types: those that acted during the exponential growth phase (i.e., they affected the maximal abundance of dinoflagellates and dinospores); and those that contributed to the elimination of dinoflagellates and dinospores once their maximal cell concentrations were achieved. For that, we evaluated whether the removal of a particular model component resulted in an increase in maximal abundances of dinoflagellates and/or dinospores. At the same time, we estimated the percentage of loss caused by parasites and/or grazers during the demise of the blooms using the loss terms of the differential equations and estimated the relative importance of parasites and grazers for the demise of blooms under eutrophic conditions (36 μM nitrate). In both cases, the intensity (%) of the negative effect was estimated by comparing the maximum abundance of the affected component in the presence and absence of the tested component under a low and a high proportion of other phytoplankton (10^4^ and 10^8^ cells L^–1^, respectively). In all simulations, the proportion between nanophytoplankton and diatoms was set at 99:1. Initial abundances of the other components were as follows: dinoflagellates = 250,000 cells L^–1^, microciliates = 3,200 cells L^–1^, rotifers = 20 ind L^–1^, dinospores = 125,000 cells L^–1^.

Finally, grazing on dinospores by rotifers was simulated under increasing nanophytoplankton concentrations (10^4^, 10^5^, 10^6^, 10^7^, 10^8^ and 10^9^cells L^–1^). To remove possible interference related to the release of dinospores from infected hosts and nanophytoplankton growth, simulations were carried out in the absence of hosts over a period of 24 h. In all simulations, dinospore and rotifer abundances were 125,000 cells L^–1^ and 20 ind L^–1^, respectively. At the same time, we assessed how two nanophytoplankton concentrations (10^5^ and 10^7^ cells L^–1^) affected the consumption of dinospores by rotifers during 30-day simulations under eutrophic condition (36 μM nitrate). Initial abundances of other components of the plankton community were as follows: dinoflagellates = 250,000 cells L^–1^, diatoms = 29,000 cells L^–1^, microciliates = 3,200 cells L^–1^, rotifers = 20 ind L^–1^, dinospores = 125,000 cells L^–1^. Simulations started without the presence of infected dinoflagellates.

All the simulations were performed using Matlab (MathWorks). Scripts are available on request from the first author.

## Results

**Fig 1:**
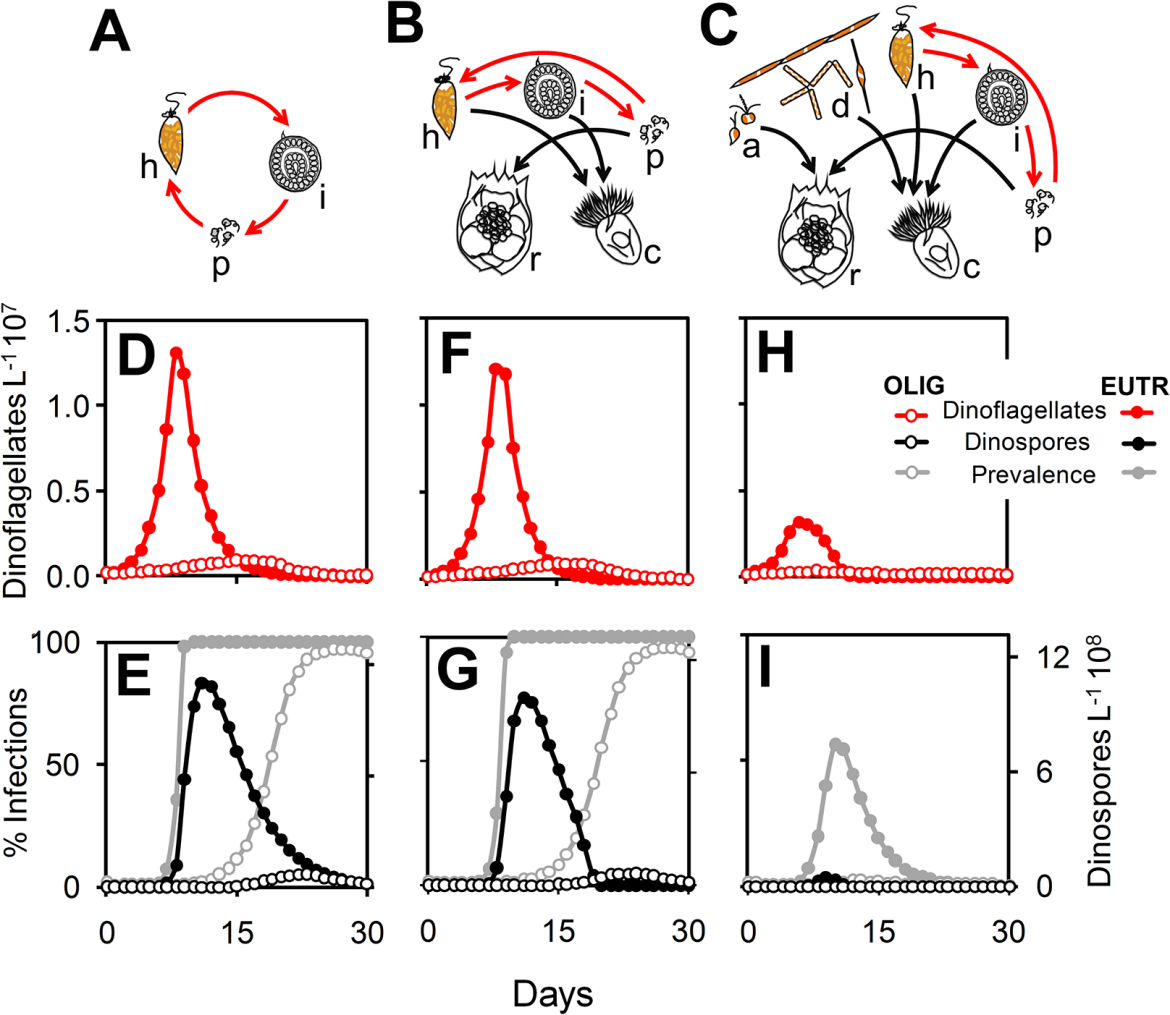
Interplay between nutrient concentration and plankton community complexity on parasite-host dynamics. (A–C) Different scenarios representing an increasing degree of community complexity: parasites and dinoflagellates only (A), parasites, dinoflagellates, and grazers (B), and parasites, dinoflagellates, grazers, and phytoplankton other than dinoflagellates (C); h = uninfected dinoflagellates, i = infected dinoflagellates, a = nanophytoplankton, d = diatoms, p = dinospores (parasite free-living stages), c = microciliates, r = rotifers. (D–I) Temporal dynamics of dinoflagellates (cells L^–1^10^6^), dinospores (cells L^–1^10^9^) and prevalence (% of infection) obtained from 30-day simulations under oligotrophic (OLIG) and eutrophic conditions (EUTR) (1 and 36 μM nitrate, respectively).

The effect of oligotrophic and eutrophic conditions (1 and 36 μM nitrate, respectively) on host-parasite dynamics were simulated under three scenarios representing an increasing degree of plankton community complexity. Without grazers (scenario 1; Fig 1A), simulations resulted in much higher abundance of dinoflagellates and dinospores in eutrophic condition than those observed under low nitrate concentration (Fig 1D and 1E). Although the prevalence (% of infection) reached almost 100% in both nitrate concentrations, a delay was observed in the timing of the parasite-host dynamics under low nitrate concentration (maximal prevalence observed on days 9 and 17 in eutrophic and oligotrophic conditions, respectively). Parasite-host dynamics remained relatively similar when grazers were added to the model (scenario 2; Fig 1B). The only difference was a faster decline of both dinospores and parasite prevalence in scenario 2 than in scenario 1 (Fig 1F and 1G). Addition of phytoplankton other than dinoflagellates (nanophytoplankton and diatoms) (scenario 3; Fig 1C) had a negative effect on dinoflagellate and dinospore abundances in both simulated trophic conditions. In contrast to scenarios 1 and 2, nitrate availability strongly affected parasite prevalence when other phytoplankton were present (maximal parasite prevalence of 99% and 10%, in eutrophic and oligrotrophic conditions, respectively) (Fig 1H and 1I).

**Fig 2:**
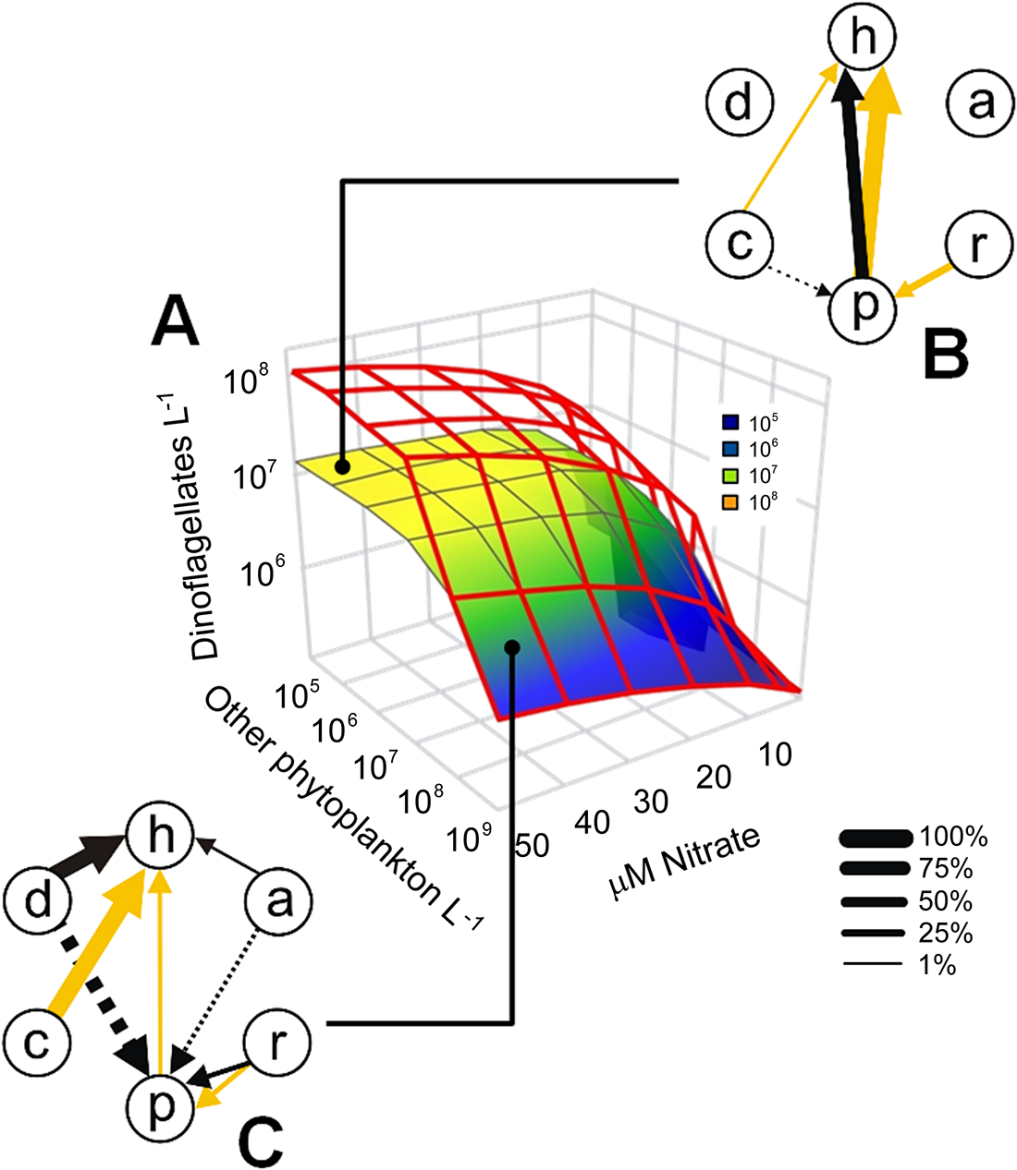
Effect of the different components of the modeled plankton community on the parasite-dinoflagellate dynamics. (A) Results of simulations assessing the effect of the initial abundance (cells L^–1^) of other phytoplankton (i.e. nanophytoplankton and diatoms) on maximum dinoflagellate abundance (cells L^–1^), in the presence (colored mesh plot) and absence (red mesh plot) of parasites, under different nitrate concentrations (μM). The composition of the plankton community was the same as in Fig 1C. (B–C) Individual relevance of the different components of the simulated plankton community that negatively affected dinoflagellates and their parasites ineutrophic conditions (36 μM nitrate) under low (B) and high (C) abundance of other phytoplankton (10^4^ and 10^8^ cells L^–1^, respectively). The proportion between nanophytoplankton and diatoms was 99:1 in all simulations. Components of the plankton community are identified by the same letters as indicated in Fig 1C. Arrow thickness indicates the intensity (%) of negative effects (only values higher than 1% are shown). Negative effects were divided into two types: those that acted during the exponential growth phase (i.e. they affected the maximum number of individuals in the population) (black arrows); and those that contributed to the elimination of dinoflagellates and parasites (yellow arrows). Dashed arrows indicate indirect negative interactions.

Dinoflagellate growth was further modeled, in the presence and absence of parasites, under varying abundance of phytoplankton other than dinoflagellates and increasing concentrations of nitrate (Fig 2A). Dinoflagellate blooms were observed only at nitrate concentrations above 20 μM and low abundance of other phytoplankton (> 10^7^ cells L^–1^). These conditions also corresponded to the highest mortality caused by parasites (maximal dinoflagellate abundance of 10^8^ and 10^7^ cells L^–1^ in the presence and absence of parasites, respectively). Under high abundance of other phytoplankton (>10^8^ cells L^–1^), dinoflagellates were not greatly impacted by an increasing nutrient availability and parasites had no effect on dinoflagellate growth.

**Fig 3:**
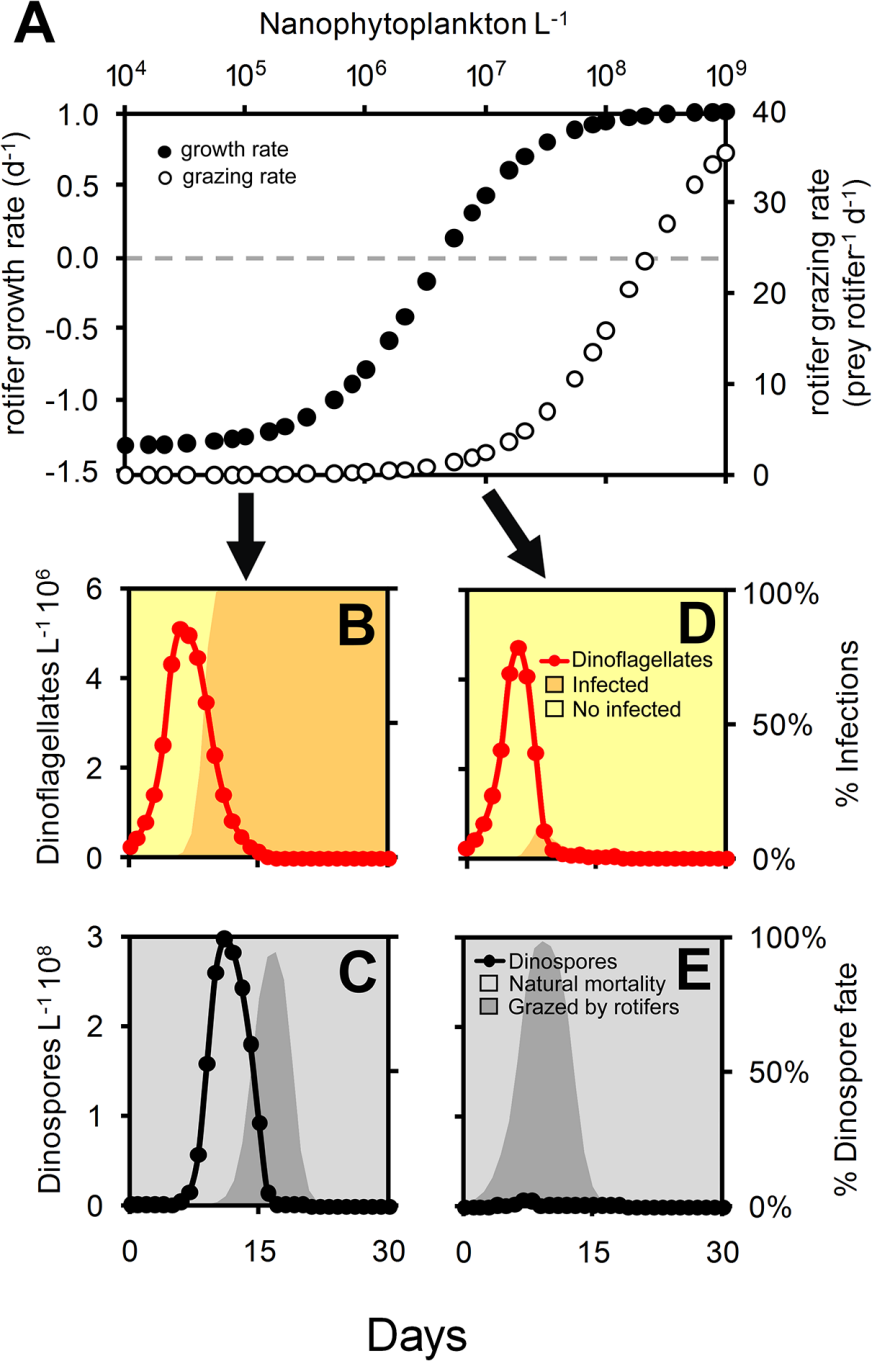
Influence of nanophytoplankton abundance on rotifer grazing of dinospores. (A) Results from simulations assessing the effect of increasing nanophytoplankton abundances (cells L^–1^) on the number of dinospores consumed by rotifers (dinospores rotifer^–1^ d^–1^) and rotifer growth rates (d^–1^). The dashed line indicates the point where rotifer growth = 0. (B–E) Effect of two nanophytoplankton concentrations, 10^5^ and 10^7^ cells L^–1^ (B–C and D–E, respectively), on the consumption of dinospores by rotifers during 30-day simulations.

An additional set of simulations showed how the different components of the modeled plankton community (i.e., other phytoplankton, grazers and parasites) can negatively affect the initiation and development of dinoflagellate blooms and/or cause the demise of their populations under eutrophic conditions (36 μM nitrate) by removing the different components of the model (one at a time). Also, these simulations indicated how parasite dinospores were affected by other components of the plankton community besides dinoflagellates. Under low abundance of other phytoplankton (10^4^ cells L^–1^; Fig 2B), parasites were the main factor controlling not only the initiation and development of dinoflagellate populations but also their extinction. Microciliates had a slight indirect negative effect on maximal dinospore abundance whereas rotifers contributed to 28% of dinospore mortality. Under high abundance of other phytoplankton (10^8^ cells L^–1^; Fig 2C), the initiation and development of dinoflagellate bloom were avoided mostly by competition with diatoms. Parasites had only a slight effect on the extinction of dinoflagellates which was caused mainly by ciliate grazing. Both diatoms and nanophytoplankton had an indirect negative effect on the maximal dinospore abundance whereas rotifers contributed up to about 84% of dinospore mortality. Increasing abundance of nanophytoplankton resulted in the increase of both growth and grazing rates of rotifers (Fig 3A). When nanophytoplankton abundance was lower than 10^4^ cells L^–1^, rotifers consumed dinospores mostly after the dinospore burst with no effect on the parasite-host dynamics (Fig 3B and 3C). On the other hand, grazing pressure on dinospores was high from the beginning of the simulations when nanophytoplankton abundance was higher than 10^7^ cells L^–1^ leading to a decrease in the number of infective dinospores (Fig 3D and 3E).

**Fig 4:**
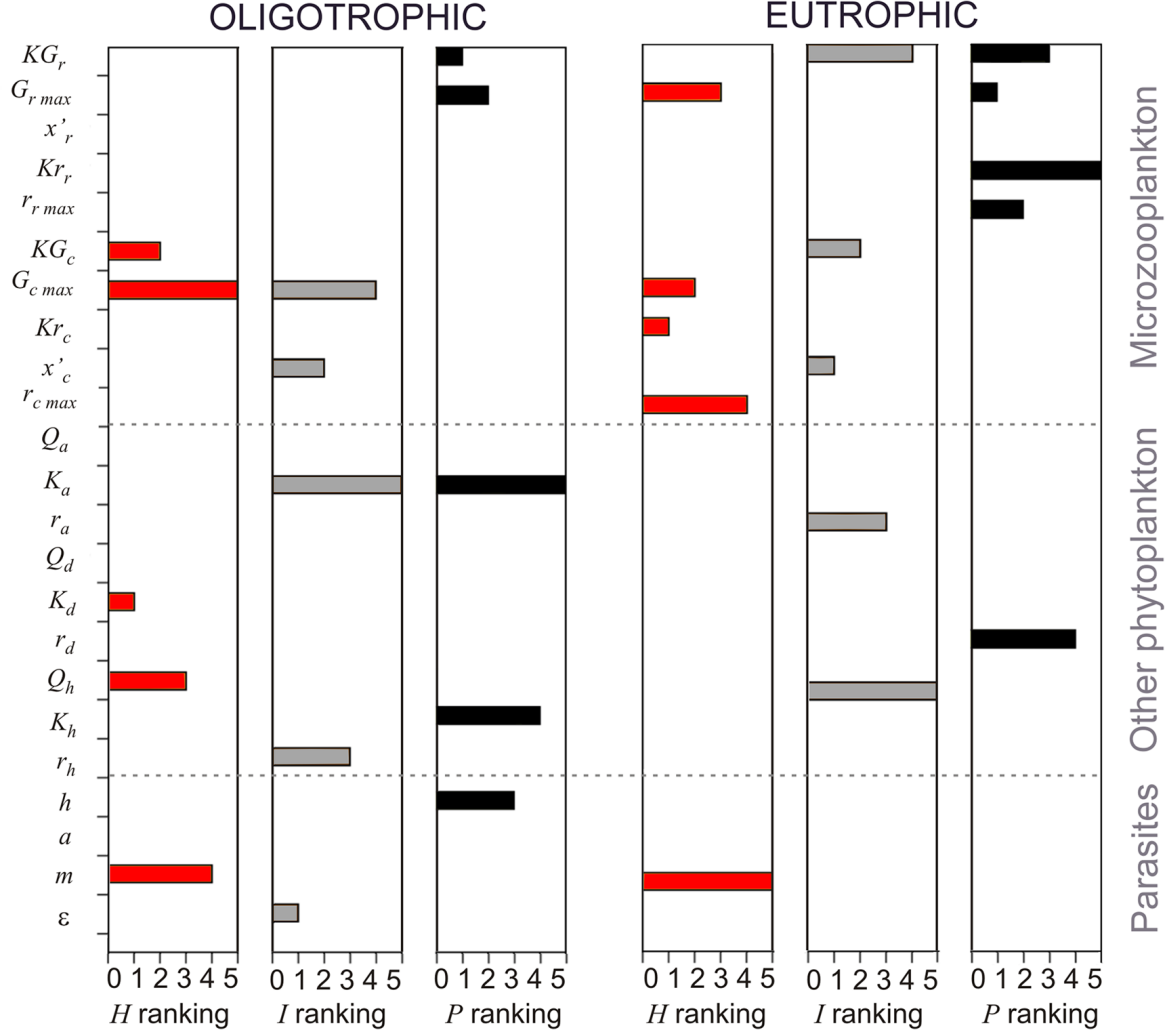
Results of Sobol’s sensitivity analysis. Ranking of first index sensitivities for the most relevant 5 parameters affecting dinoflagellate abundance (*H*; red bars), infected dinoflagellate cells (*I*; grey bars) and dinospore abundance (*P*; black bars) under oligotrophic and eutrophic conditions (1 and 36 μM nitrate, respectively). Parameters are numbered from 1 (most influencing) to 5. Meanings of parameter symbols are the same as indicated in Table 1.

Results from the global sensitivity analysis (Fig 4) indicated that, under nutrient limitation (oligotrophic condition; 1 μM nitrate), dinoflagellates were affected mainly by the half-saturation constant of diatoms. On the other hand, the half saturation constant for ciliate growth was the most important model parameter affecting dinoflagellates under high nutrient availability (eutrophic condition; 36 μM nitrate). The amount of infected dinoflagellates was greatly influenced by the number of dinospores released per infected host under oligotrophic condition whereas the prey threshold for ciliate growth was the key parameter influencing infections under eutrophic conditions. Finally, dinospore density was most affected by the half-saturation constant for rotifer grazing and the maximal ingestion rate of rotifers under oligotrophic and eutrophic conditions, respectively.

## Discussion

Eutrophication has been indicated as one of the main factors contributing to the increase in frequency and geographic distribution of HABs observed in the past decades [39]. In this context, considerable effort has been made to understand how changing nutrient concentrations and ratios result in the tendency of toxic or otherwise harmful opportunistic algal species to bloom (reviewed by [6]). However, blooms should not be considered to be exclusively regulated by abiotic factors as they are also under biological control [40]. In this study, numerical simulations indicated that parasites had greater impact on the development of dinoflagellate blooms than microciliates (as previously pointed out by [15]). Grazing by microzooplankton has been proposed as an important mechanism controlling dinoflagellate blooms since protistan grazers are capable of growing as fast as their phytoplankton preys [41]. However, the generally high prey threshold of microzooplankton organisms such as ciliates implies a large time delay in their numerical-functional responses (i.e., increase/decrease in growth and grazing rates of predators according to the total amount of available preys) [20]. The sensitivity analysis of our model indicated that dinoflagellate abundance was sensitive to changes in the values of the half saturation constant for ciliate growth (Fig 4). This is in agreement with simulations performed by Montagnes et al. [15] where ciliates were only able to control dinoflagellate blooms when values for their grazing parameters were lower than recorded in the literature. As a consequence, although microzooplankton is capable of eliminating dinoflagellate blooms once they are established [16, 42], they are not likely to be capable of preventing their initiation [20].

Parasites of genus *Amoebophrya* have been identified as potentially important biotic factors controlling dinoflagellate blooms [13, 14]. Host abundance has been proposed as the main factor affecting infective rates of these parasites [10]. This is related to the density-dependence of parasite dynamics since increasing host density enhances the risk of the host to become infected through increased encounter rates [43]. Given that each *Amoebophrya* infection produces hundreds of dinospores [12], even a small increase in the number of available hosts would result in a significant increase in the number of propagules released at any given moment of the parasite-host dynamics. Based on that, the stimulation of dinoflagellate growth resulting from increased nutrient availability would always lead to higher parasitic rates. However, the modeling approach considered in this study indicated that the effect of temporary eutrophication on the parasitic control of dinoflagellate blooms should be considered within a community context.

Nutrient availability affected parasitic control of dinoflagellates, but the magnitude of this effect was likely determined by the presence/absence as well as the relative abundance of other members of the plankton community which would exert direct and/or indirect effects on host-parasite dynamics. When host and parasites were considered independently, parasites exerted a complete control on the dinoflagellate host (Fig 1D and 1E), as expected from previous results of both experimental and modeling studies [12, 18]. We expected that the addition of grazers to this simple system would affect host-parasite dynamics, since it has been shown that grazing on the free-living stages of parasites could significantly diminish parasite prevalence in plankton systems [9, 17, 44]. However, while grazers by themselves were not important (Fig 1F and 1G), differences in both parasite prevalence and dinospore density related to nutrient concentrations were greatly magnified by the addition of other phytoplankton (Fig 1H and 1I).

Numerical simulations indicated that although nutrient availability importantly affected dinoflagellate growth (no bloom was formed at nitrate concentrations below 20 μM) (Fig 2A), the predominant biotic mechanism controlling dinoflagellate populations depended strongly on the relative abundance of the different phytoplankton groups at the beginning of the simulations. When diatoms and nanophytoplankton were dominant, the formation of dinoflagellate blooms under high nutrient concentrations was impeded mostly by resource competition for nutrients (mainly with diatoms) (Fig 2B), related to higher growth rates and low half-saturation constant for nitrate uptake usually observed in diatoms [45]. This is in agreement with the results of the sensitivity analysis that indicated the half saturation constant for nitrate uptake of diatoms as the most relevant parameter of the model affecting dinoflagellate abundance (Fig 4). Parasites importantly reduced the size of dinoflagellate populations, but caused its elimination only when dinoflagellates dominated phytoplankton assemblages (Fig 2C).

The indirect negative effect of diatoms and nanophytoplankton on free-living parasite stages was an unexpected result of these simulations. By outcompeting dinoflagellates for nutrient uptake, other phytoplankton limited the number of hosts available for infection, which in turn decreased the total amount of released dinospores. Interestingly, a high abundance of other phytoplankton also determined ciliates (and not parasites) to be the main cause for the elimination of dinoflagellates. Simultaneously, the nanophytoplankton stimulated predation on the parasites by affecting numerical-functional responses of rotifers (Fig 3A). When nanophytoplankton abundance was lower than 10^7^ cells L^–1^, the total amount of available prey was below the threshold for rotifer growth. As a consequence, rotifers consumed dinospores mostly after their maximal abundance was observed (and most of the hosts were killed) with no effect on the host-parasite dynamics (Fig 3B and 3C). On the other hand, when the abundance of nanophytoplankton was higher than 10^7^ cells L^–1^, grazing pressure on dinospores was high from the beginning of the simulations leading to a decrease in the number of dinospores able to establish infections (Fig 3D and 3E). This is very relevant considering that the sensitivity analysis indicated that dinospore abundance was mostly affected by parameters related to rotifer grazing (Fig 4). Predation on free-living parasite stages is an important biotic factor affecting parasite transmission [46]. However, previous experimental evidence is contradictory in that the presence of predators could lead either to an increase or decrease in parasite prevalence [47, 48]. Our results suggest that the presence of alternative prey (potentially affecting the numerical-functional response of predators) should also be taken into account to understand the conditions under which predators affect host-parasite dynamics.

Parasite-mediated interactions are widely known to significantly affect population structure, trophic relationships and energy flow within ecological communities [49]. Less frequent is the perception that parasites are in turn affected by interactions with other species besides their focal hosts. The ‘dilution effect hypothesis’ suggests that the net effect of increasing species richness reduces the risk of certain infectious diseases in ecological communities [50]. Although initially focused on how the presence of a diverse assemblage of relatively inefficient hosts would reduce parasite transfer to competent hosts [51], it is now clear that other mechanisms related to non-host species may also act as buffers against parasite transmission [47, 52]. Here, we provide evidence that diverse phytoplankton assemblages that are functionally different from the host (e.g. diatoms and nanophytoplankton) may have an important indirect dilution effect on the parasitic control of dinoflagellate blooms either by competing for resources with dinoflagellates (and thus limiting the number of hosts available for infection) or by stimulating microzooplankton grazing on free-living parasite stages. We therefore speculate that parasites are likely more efficient as controlling agents of dinoflagellates during monospecific blooms. Forthcoming experimental research should consider possible interplay between parasitism and strategies frequently used by dinoflagellates to increase their competitive performance (e.g., mixotrophy and allelopathy [53, 54]).

The assessment of the relevance of eutrophication on HAB dynamics should take into account that plankton communities as a whole and not only HAB species are impacted by nutrient input, resulting in changes of the biotic interactions [40]. The modeling approach proposed in this study indicated that although nutrient availability promotes both the growth of dinoflagellates and the development of their protozoan parasites, the efficacy of parasitic control of dinoflagellate blooms under temporary eutrophication depends strongly on the structure of the plankton community at the moment when the nutrient pulse occurs. The modeling outcomes further underscore the importance of accounting for indirect interactions when assessing the relative importance of bottom-up and top-down factors on HAB dynamics.

## Supporting Information

S1 Appendix Plankton structure used for the formulation of the model. Section S1–1. Determination of the variables. Section S1–2. Nitrate concentrations and composition of the plankton community.
(DOC)

S1 Fig Outputs of the 30-d numerical simulations for phytoplankton other than dinoflagellates (A–B) and microzooplankton (C–D). (PNG)
