# Supplementary material for: Significance of Plankton Community Structure and Nutrient Availability for the Control of Dinoflagellate Blooms by Parasites: A Modeling Approach
Source: PLoS One. 2015 Jun 1;10(6):e0127623. doi: 10.1371/journal.pone.0127623 (PMC4452582; doi:10.1371/journal.pone.0127623)
Supplement: S1 Appendix — Section S1–1. Determination of the variables. Section S1–2. Nitrate concentrations and composition of the plankton community. (DOC) [file pone.0127623.s001.doc]

**S1 Appendix**

**Planktic structure used for the formulation of the model**

**1. Determination of the variables**

The initial values of variables used in the model were based on the specific composition of the eukaryotic plankton community at the beginning of a mesocosm experiment performed in Thau Lagoon coastal waters (southern France). This experiment was performed during autumn 2009 at the Mediterranean platform for Marine Ecosystem Experimental Research (MEDIMEER, <http://www.medimeer.univ-montp2.fr/>) located at Thau Lagoon in the South of France (43°24’49” N, 3°41’19” E) . A nutrient pulse was obtained by adding waters from the Véne River to the mesocosms in order to simulate a temporary eutrophication following river floods, phenomena that are usually observed at the Thau Lagoon . In order to obtain the initial values of the components of the plankton community used in the model (Table 1), lagoon water samples were taken at the first day of the experiment before the after the addition of the river water. Samples for the determination of NO_3_+NO_2_ were taken before and following the addition of the river water. All samples were taken in two replicates, except for *Amoebophrya* determination (sampled once).

**Nitrate concentrations.** Samples (80 ml) were filtered onto pre-combusted glass-fiber filters (Whatman GF/F, 27 mm) and stored in the freezer (–20°C) until analysis. Concentrations of nitrate (NO_3_+NO_2_) was determined using an automated colorimeter (Skalar) with standard nutrient analysis methods .

**Microscopic counts.** Microphytoplankton (> 20 µm) was enumerated from 125 ml samples fixed with 2% Lugol’s solution. Quantifications were performed by the Utermöhl method under an inverted microscope (Nikon Eclipse TS100) after overnight sedimentation in 10 ml columns. Units (cells or colonies) were quantified in random fields under 20× magnification until at least 100 units of the dominant species were enumerated (*p* < 0.05) . For microciliates, samples (125 ml) were fixed in 6.4% Lugol’s solution. Subsamples (100 ml on days 1 and 2, and 10 ml on days 4 and 6) were left to settle in Utermöhl chambers for 24 h and then counted under 40× magnification with an inverted microscope (Olympus IX70). At least 100 ciliates were quantified using the entire Utermöhl chamber when ciliate density was low, or half of the chamber when it was high. For rotifers, 60 L samples were filtered through a 60-µm sieve and fixed in 50 ml of buffered formalin (4%). Individuals were taxonomically enumerated using a Leica MZ6 dissecting microscope.

**Flow cytometry analyses.** The qualitative analysis of nanophytoplankton (> 20 µm) from Lugol's samples showed that this size fraction in Thau lagoon during the mesocosm experiment was made up mostly by cells <10 µm what allowed their quantification by flow cytometry. Frozen fixed samples (2% formaldehyde, frozen at –196°C and stored at –80°C) were thawed and analyzed using a FACSCalibur flow cytometer (Becton-Dickinson, Franklin Lakes, NJ, USA) equipped with an air-cooled laser providing 15 mW at 488 nm with the standard filter set-up. Nanophytoplankton cells were distinguished on the basis of optical characteristics (FSC Forward SCatter, and SSC Side SCatter) and natural red fluorescence emissions (chlorophyll *a*, wavelength > 650 nm). A mixture of fluorescent beads of 0.96 and 1.8 µm diameters (Molecular Probes Inc., Eugene, OR, U.S.A.) was added to each sample to normalize the optical parameters. TruCount beads (Becton Dickinson) were used to determine the analyzed volumes of each sample. Duplicate subsamples were run for 5 min at high flow rate. Data were analyzed using CellQuest Pro (Becton-Dickinson).

**Parasites.** *Amoebophrya* parasites were detected and enumerated by fluorescent *in situ* hybridization coupled with tyramide signal amplification (FISH-TSA) as described by Alves-de-Souza et al. . Dinospores (free-living parasite stages) were counted with the 100× objective in 20 randomly chosen microscopic fields. For the estimation of parasite prevalence (i.e., number of infected hosts), 100 dinoflagellate specimens (infected or not) were observed .

**2. Nitrate concentrations and composition of the plankton community**

At the beginning of the experiment, lagoon waters were characterized by low concentrations of NO_3_+NO_2_ (1±0.01 µM) which increased (36±2.12) after river water addition. Microphytoplankton was characterized by low total cell abundance (1.76±0.05×10^5^ cells L^–1^) and the predominance (99%) of dinoflagellates (made up almost exclusively by *Prorocentrum triestinum*). Main diatom species were *Leptocylindrus minimus*, *Leptocylindrus danicus*, *Cylindrotheca closterium, Pseudo-nitzschia* spp*.* and *Thalassionema nitzschioides*. Nanophytoplankton abundance was 1.9±0.6×10^6^ cells L^–1^. Qualitative analysis of Lugol's samples indicated that this size fraction was composed mainly by cryptophyceans, haptophyceans and prasinophyceans. Microciliate and rotifer abundances were 3.2±0.1×10^3^ cells L^–1^ and 20±0.5 ind L^–1^, respectively. Rotifers were composed mainly by species of the genus *Brachionus*. *Amoebophrya* sp. prevalence of infections on *P. triestinum* was 2% whereas dinospore abundance was 11.6±0.2×10^4^ cells L^–1^.

References

1. Fouilland E, Descolas-Gros C, Collos Y, Vaquer A, Souchu P, et al. (2002) Influence of nitrogen enrichment on size-fractionated in vitro carboxylase activities of phytoplankton from Thau Lagoon (Coastal Mediterranean Lagoon, France). Journal of Experimental Marine Biology and Ecology 275: 147-171.

2. Pecqueur D, Vidussi F, Fouilland E, Le Floc’h E, Mas S, et al. (2011) Dynamics of microbial planktonic food web components during a river flash flood in a Mediterranean coastal lagoon. Hydrobiologia 673: 13-27.

3. Fouilland E, Trottet A, Bancon-Montigny C, Bouvy M, Le Floc'h E, et al. (2012) Impact of a river flash flood on microbial carbon and nitrogen production in a Mediterranean Lagoon (Thau Lagoon, France). Estuarine, Coastal and Shelf Science 113: 192-204.

4. Tréguer P, Le Corre P (1975) Handbook of seawater nutrient analyses. Autoanalyser II Technicon user guide. Brest: Université Bretagne Occidentale. 110 p.

5. Utermöhl H (1958) Zur vendlhommung der quantitativen phytoplankton. Methodik Int Ver Theoret Agur Limnol 9: 1-38.

6. Uherlinger V (1964) Étude statistique des methods de dénobrement planctonique. Archives de Science 17: 121-223.

7. Lund JWG, Kipling C, Lecren ED (1958) The inverted microscope method of estimating algal number and the statistical basis of estimating by counting. Hydrobiologia 11: 143-170.

8. Alves-de-Souza C, Varela D, Iriarte JL, González HE, Guillou L (2012) Infection dynamics of Amoebophryidae parasitoids on harmful dinoflagellates in a southern Chilean fjord dominated by diatoms. Aquatic Microbial Ecology 66: 183-187.

9. Coats DW, Bockstahler KR (1994) Occurrence of the parasitic dinoflagellate *Amoebophrya ceratii* in Chesapeake Bay populations of *Gymnodinium sanguineum*. Journal of Eukaryotic Microbiology 41: 586-593.
