# Supplementary figures and images for: Significance of Plankton Community Structure and Nutrient Availability for the Control of Dinoflagellate Blooms by Parasites: A Modeling Approach
Source: PLoS One. 2015 Jun 1;10(6):e0127623. doi: 10.1371/journal.pone.0127623 (PMC4452582; doi:10.1371/journal.pone.0127623)

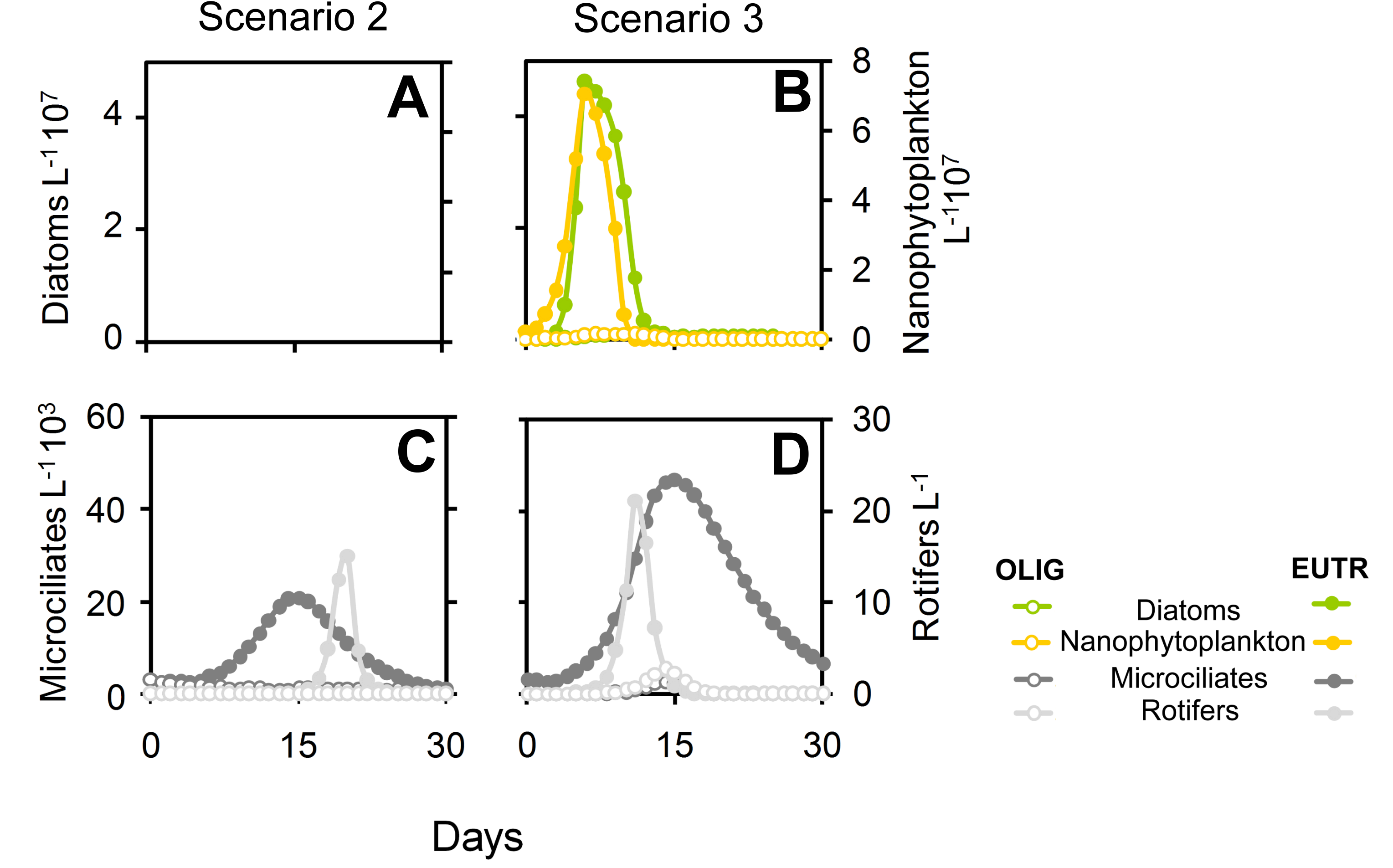

Supplement: S1 Fig — (PNG) [file pone.0127623.s002.png]
